# Barriers to and Enablers of Preventive Sexual and Reproductive Health Care Among Women Seeking Asylum in Melbourne, Victoria: A Qualitative Study

**DOI:** 10.3390/ijerph22121836

**Published:** 2025-12-08

**Authors:** Natasha Davidson, Karin Hammarberg, Jane Fisher

**Affiliations:** Global and Women’s Health, School of Public Health and Preventive Medicine, Faculty of Medicine Nursing and Health Sciences, Monash University, Melbourne, VIC 3004, Australia

**Keywords:** asylum seekers, prevention, qualitative, sexual and reproductive health, women

## Abstract

Women seeking asylum experience markedly poorer health outcomes than refugees, other migrants, and host populations, with sexual and reproductive health (SRH) needs that are complex and multidimensional. This qualitative study explored the barriers to and enablers of accessing preventive SRH care among women seeking asylum in Australia. Between March 2022 and September 2023, semi-structured interviews were conducted with twelve women from eight countries. Using a socioecological framework, analysis revealed that access to preventive SRH care is shaped by intersecting factors at individual, interpersonal, community, and policy levels. Key barriers included limited knowledge of preventive care, psychosocial and financial constraints, fragmented health services, and restrictive immigration policies. Enabling factors included culturally concordant care, continuity with trusted general practitioners, and supportive community and social relationships. The findings underscore how structural and relational factors intersect to influence SRH access and highlight the need for coordinated, multi-level strategies to promote equitable SRH care for women seeking asylum in Australia.

## 1. Introduction

The United Nations High Commissioner for Refugees defines asylum seekers as “individuals who have sought international protection and whose claims for refugee status have not yet been determined”. Through Australia’s humanitarian program, refugees are granted permanent residency and unrestricted access to government-funded services, facilitated by the Humanitarian Settlement Program [1]. By contrast, those who claim asylum in Australia have restricted access to health and welfare services [2]. As of 2021, an estimated 35,800 asylum seekers were living in Australia, many on bridging visas [3]. Victoria is the second most populous state in Australia, with over one-quarter of the national population (7 out of 25 million). A large proportion of people seeking asylum live in this state. The majority of asylum seekers residing in Australia come from Afghanistan, Iran, Bangladesh, Sri Lanka, and Iraq, with between 11,000 and 20,000 living in Victoria [4]. Most of those living in the community are on bridging visas granting them limited or no rights to work and limited access to health care [2]. Not yet recognised as refugees, they face uncertainty as they await the outcome of their claim for refugee status, fear being returned to their country of origin, and many experience unemployment, homelessness and poor mental health [5,6,7].

The Australian health care system is a combination of public and private services and insurance arrangements. Medicare, Australia’s public health insurance system, grants universal access to medical and pharmacy services and hospital treatment [8]. A key barrier to the health care of some asylum seekers is their ineligibility to access Medicare, resulting in differential health care, lower service utilisation and poorer health care outcomes [9].

Upon arrival in Australia, people seeking asylum may be placed either in community detention or in immigration detention, depending on a range of factors including visa status, mode of arrival, and government policy at the time. Immigration detention confines people in secure facilities, while community detention allows them to live within the community under certain conditions and supervision [10]. People seeking asylum who are held in immigration detention face severe challenges including restricted freedom [11]; psychological distress from prolonged detention [12]; social isolation; limited access to high quality health care; and women in particular may experience violence including intimate partner violence [13].

People seeking asylum have significantly poorer health than refugees, other immigrants and host country populations [14,15,16]. Complex health problems experienced by people seeking asylum have been documented in a number of high-income country settings, including Australia [17,18]. Despite this, asylum seekers can experience multilevel barriers is accessing and utilising health services. A global systematic review of asylum seeker health care in high-income countries identified some of the key barriers which include affordability, lack of familiarity with the health care system, perceived effectiveness of health services, medical mistrust, linguistic and cultural barriers and discrimination [17]. Substantial evidence exists that asylum seekers are at higher risk of post-traumatic stress disorder (PTSD) due to experiences in their country of origin and during transit [18]. Evidence from the UK and US suggests that some asylum seekers avoid accessing government-run health services due to fears of imprisonment, deportation, and potential negative impacts on their asylum claims [19].

Sexual and reproductive health (SRH) problems are multifaceted and often intertwined with their broader health and social challenges. Research conducted in other countries suggest women seeking asylum have distinct heath care needs, including relating to SRH [20]. Women seeking asylum can experience difficulties accessing contraceptive services due to legal, financial, and language barriers, leading to unintended pregnancies [21]. Limited access to preventive screenings for cervical and breast cancer can delay diagnosis and treatment of these conditions [22]. In addition, many women seeking asylum have experienced sexual violence and exploitation in their home countries or in transit, contributing to significant physical, psychological trauma and poor mental health [23]. The impact of sexual violence often leads to mental health issues such as PTSD, depression, and anxiety, which can be compounded by ongoing stress and uncertainty [23]. In Australia, SRH challenges among asylum seekers are multifaceted and include: policy limitations on accessing publicly funded primary health care [24]; inconsistent and fragmented care [25]; and barriers in the transition from asylum seeker–specific health services to mainstream primary care [26]. Given these challenges, it is essential to explore the factors shaping preventive SRH access for asylum-seeking women to inform equitable health care policies and improve service delivery.

Despite the growing attention to the health needs of refugees, there remains limited research focusing specifically on women seeking asylum in Australia, their access to preventive SRH care or their views on the barriers to and enablers of care. This study addresses this gap by exploring the experiences of women seeking asylum through a qualitative lens, using the socioecological model to capture influences at multiple levels. By examining these perspectives, the study provides novel insights into barriers to and enablers of preventive SRH care and aims to inform strategies for improving access and health equity for this underserved population.

The aim of this study was to establish the barriers to and enablers of accessing preventive SRH for women seeking asylum living in Melbourne. In this study, preventive SRH includes contraceptive care, cervical screening, information about human papillomavirus (HPV) vaccination and breast screening.

## 2. Materials and Methods

A qualitative descriptive design using semi-structured interviews was conducted. Semi structured interviews are suitable for exploring complex behaviours, opinions, and emotions [27].

### 2.1. Study Setting and Context

The study was conducted in, Melbourne, Australia. Large populations of people seeking asylum live within the local government area of Brimbank where the research was undertaken. In 2021, approximately 50% of the population in this region was born overseas and about 55% speak a language other than English at home [28].

### 2.2. Inclusion Criteria

Women aged over 18 years who were seeking asylum were eligible to participate.

### 2.3. Participants and Recruitment

Flyers with information about the study’s purpose, eligibility criteria and the researcher’s contact details were distributed widely to non-government charitable organisations responsible for resettlement of people seeking asylum. Multiple recruitment strategies were used, including outreach through services, professional networks, and word of mouth. Consequently, women were likely informed of the study via a service provider or community leader. These methods may have missed women who were truly ‘hard to reach,’ such as those not connected to services or social networks. The first author introduced herself to the key contact person at these organisations who then took responsibility for recruitment. The researcher provided a clear and transparent explanation of the recruitment process to the contact person detailing how potential participants should be identified, approached and recruited. By email or telephone, the researcher then described the study to potential participants and explained that it was their choice to participate or not. Potential participants were asked to contact the researcher who arranged an interview. The choice to participate was reiterated again prior to the interview being conducted. Convenience sampling was used based on women’s availability and willingness to participate. Oral and written consent was obtained before the interview.

Women seeking asylum in Australia constitute a highly marginalised and hard-to-reach population. Consequently, pragmatic considerations and ethical sensitivity informed the final sample size. Considerable efforts were made to engage this group through trusted community networks, service providers, and advocacy organisations. Despite these challenges, twelve women consented to participate, representing a meaningful and diverse sample given the significant barriers to access, including transience, distrust of institutions and authority, and concerns about confidentiality impacting their visa status.

### 2.4. Conceptual Framework

McLeroy’s socioecological model was used as a framework for this study [29]. The model emphasises that health behaviours and outcomes are influenced by multiple levels of factors, from the individual to broader societal contexts [30]. By utilising the multiple layers of the socioecological model, the potential challenges in the provision of preventive SRH care, barriers to and enablers of care experienced by women seeking asylum may be comprehensively explained.

### 2.5. Data Source

An interview guide was developed based on findings of a systematic review [31] and the researchers refugee health experience. It included questions relating to contraceptive care, cervical screening tests, HPV vaccination and breast screening, exploring awareness, knowledge and utilisation of these preventive measures; barriers to and enablers of accessing preventive SRH care; and sources of information about preventive SRH.

Davidson and colleagues (2022) conducted a systematic review [31] and identified themes relating to SRH care access including knowledge, awareness, perceived need for and use of preventive SRH care; language and communication barriers. This evidence directly informed the interview questions; “Have you heard about women’s health problems? [contraceptive care, cervical screening, HPV vaccination and breast screen], “Have you had a… [cervical screening, HPV vaccination and breast screen,]” and “Do you know why it’s done [cervical screening, HPV vaccination and breast screen]”?

### 2.6. Reflexivity—Positioning the Researcher

Reflexivity involves critically examining how a researcher’s social, cultural, and professional contexts shape the research process [32]. The first author’s (ND) extensive experience in refugee health, both internationally and within Australia, has significantly influenced her motivation and approach to this study. As a Registered Nurse with over thirty years of experience across tertiary hospitals and primary health care settings, she has developed a deep understanding of health disparities affecting vulnerable populations. She has undertaken humanitarian aid postings with the International Committee of the Red Cross, in Tanzania, Myanmar, and Banda Aceh, Indonesia, together with extensive work and travel in low- and middle-income countries, which has further informed this perspective.

The first author’s professional and humanitarian background may have influenced the interview process in several ways. Her extensive experience working with refugee populations likely facilitated rapport and trust with women, enabling open and in-depth discussions. At the same time, her familiarity with refugee health challenges may have shaped the direction of questioning and interpretation of participants’ narratives. To address this, she engaged in ongoing reflexive practice, maintaining awareness of her assumptions and striving to ensure that participants’ voices and perspectives remained central to the analysis.

### 2.7. Data Collection Procedure

Between March 2022 and September 2023 face to face semi-structured interviews were conducted. Participants not fluent in English were provided with a NAATI (National Accreditation Authority for Translators and Interpreters) accredited interpreter [33]. Interviews were conducted in a private room, free from interruptions, with the researcher, interpreter and interviewee present. Familiar locations were chosen, maximising the likelihood participants felt safe, comfortable, and at ease during interviews. Prior to the interview participants were assured that their responses would be anonymous and given the option of choosing their own pseudonym or having a pseudonym chosen by the researcher. This pseudonym was used in data coding, analysis and presentation of findings. Interviews were audio recorded and the English language data transcribed verbatim by ND.

Data collection and analysis occurred concurrently. Interviews were conducted until thematic saturation was reached, that is, when no new themes or insights emerged from the data [34]. After approximately eight interviews, recurrent patterns were evident, and subsequent interviews confirmed these existing themes. Saturation was assessed informally through ongoing team discussions and review of coded transcripts, consistent with established qualitative research practices [35].

### 2.8. Rigor and Trustworthiness

Several strategies were employed to enhance the rigour of this study and ensure credibility, dependability, confirmability, and transferability of the findings. Credibility was supported through prolonged engagement with women, informal member checking during interviews, and regular supervisory discussions to ensure interpretations reflected womens’ perspectives. Dependability was enhanced by maintaining a detailed audit trail of recruitment procedures, analytical decisions, and coding processes, enabling transparency and consistency throughout the study. Confirmability was achieved through reflexive journaling to monitor potential researcher bias, combined with peer review with JF and KH coding and conducting theme development to ensure findings were grounded in womens’ accounts. Transferability was promoted by providing rich descriptions of womens’ contexts and experiences, including their diverse countries of origin, age ranges, and migration histories, allowing readers to assess the relevance of findings to other settings.

### 2.9. Data Management and Analysis

Data management, coding and analysis were undertaken using in NVivo 12 software (QSR International, 2023) and analysed using reflective thematic analysis approach [36]. A combination of both deductive and inductive approaches was used for coding. Pre-defined codes were generated based on questions in the interview guide. Throughout analysis, the coding underwent several revisions as themes were identified on the basis of responses. Throughout revisions, ND crossed-checked the coding with JF and KH to ensure consistency within and between interviews. Coding was performed independently by ND for each transcript by close line by line reading. JF and KH reviewed a sample of the coded transcripts for consistency. No major disagreements between authors over theme identification took place. Once this process was complete, patterns across interviews were identified and presented as themes and subthemes. A socioecological framework which accounts for the multifaceted interrelationships between individual, interpersonal, community and policy level factors was used to categorise and interpret findings [29].

### 2.10. Ethics Approval

The study was approved by Monash University Human Research Ethics Committee (Project ID 28080).

## 3. Results

### 3.1. Participant Characteristics

Of the 14 women seeking asylum who were approached, 12 were interviewed. Two women declined to be interviewed. Six interviews were conducted in English and six using Arabic, Persian and Tamil interpreters. Five interviews were held at a local Public Library, three in participants’ homes and one each in a local café, a leisure centre, a sports oval and a university office. The mean duration of interviews was 60 min (range, 43 to 97 min). The mean age was 36 years (SD = 5.7). The mean duration of residence in Australia was 7.8 years (SD = 3.4). Participants’ sociodemographic characteristics are shown in Table 1. Individual participant characteristics are shown in Table 2.

### 3.2. Levels of Influence

**Figure 1:**
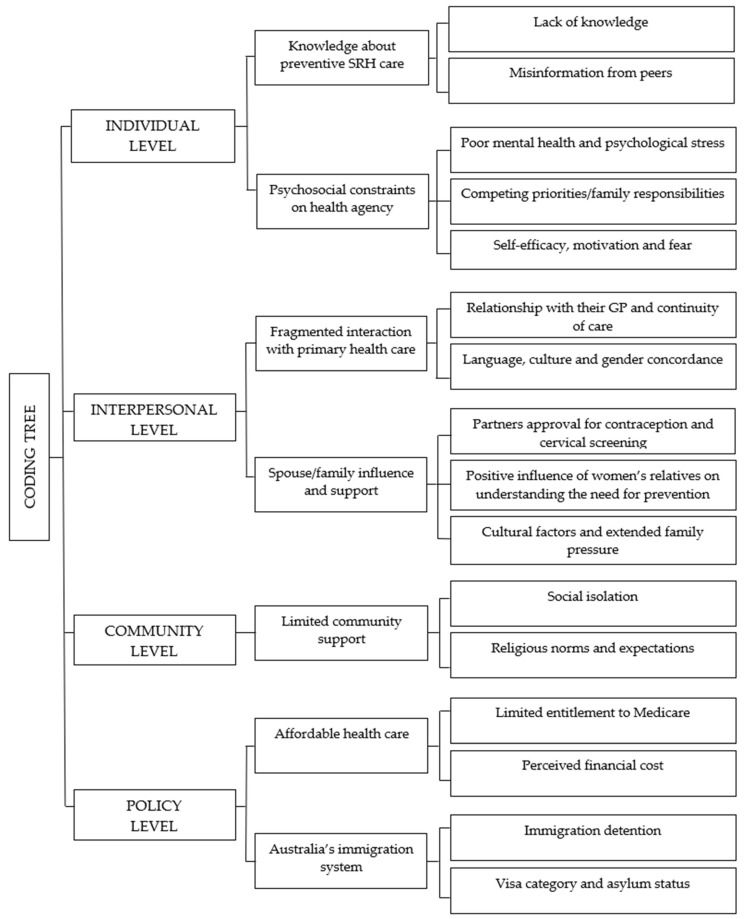
Coding Tree.

Barriers to and enablers of accessing preventive SRH care were identified at four levels of influence: individual, interpersonal, community and policy. Across four levels, seven major themes and 16 subthemes were identified and are summarised in the coding tree (Figure 1). Findings at each level of influence are summarised below.

### 3.3. Individual Level

Theme: Knowledge about preventive SRH care

#### 3.3.1. Lack of Knowledge

Overall knowledge and awareness of preventive care varied by topic. Drawing on experiences in their countries of origin, most women demonstrated awareness of breast and cervical screening, with comparatively lower levels of familiarity reported in relation to HPV vaccination or contraception.

*“Yes, [cervical] screening is for anyone it gets cancer earlier, we can diagnose with the pap smear or any other kind of vaginal disease or anything from this we can early detect.”* [Breast screening] That’s also early screening test for detecting breast cancer that’s a really good opportunity (Shiryani, 46)

A minority of women reported having no prior awareness of contraception or HPV vaccination since arrival in Australia.

*“I don’t know anything about contraception, or I didn’t know anything about it back in my home country and I learned about that only after I had the baby, when the baby was 6 weeks old, I had to go and see a GP and that’s the time they asked these questions and talked about contraception, that’s the first time I heard about that before that I didn’t know anything about contraception.”* (Vijitha, 34)

When asked about their awareness and understanding of HPV vaccination, several women indicated that they had no prior knowledge of it.

*“No I don’t know about this [HPV Vaccination]… and I’ve never heard about it in Lebanon.”* (Iman, 37)

#### 3.3.2. Misinformation from Peers

There were mixed results about the impact of peer influence on undertaking preventive SRH, with some women receiving misinformation from peers, notably concerning the side effects of contraception.

*“…people [women] that are not aware of what it is and just listening to misinterpretation, for example because some people said that if you take birth control you have cancer,… and like no it doesn’t mean that. Birth control doesn’t do that, it doesn’t cause cancer. It stops you from getting pregnant. So, it’s the misunderstanding and just listening to people that don’t know much about these things and maybe they are just spreading wrong information to others.”* (Afidj, 35)

Women’s decisions around cervical screening were also shaped by the views and experiences of peers and social networks.

*“there are some other cousins or friends who come here and they say they are going for the cervical screen and I asked them what it was all about. Then they said I don’t need to go.”* (Vijitha, 28)

Women’s contraceptive decisions were shaped by perceived risks, such as fear of weight gain and infection, often reinforced by the experiences and advice of friends and family.

*“Yes, my sister and my friends all stopped because they are gaining weight so they stopped the implants. And some people are afraid of the IUD because people will get an infection for using IUD so they stop. They are afraid to use it.”* (Jen, 34)

Theme 2: Psychosocial constraints on health agency

The impact of poor mental health on access to preventive SRH care of was compounded by the competing priorities, including low prioritisation of women’s own health needs, family caring responsibilities and finding employment.

#### 3.3.3. Poor Mental Health and Psychological Stress

Many women described how psychological distress and low mood diminished their motivation to prioritise their own health, including participation in breast and cervical screening

*“…I have seen some people here and back in Iran and I think it [barriers to accessing screening] is linked to their low mood and their mental health. Like they are drowning in their problems, they forget about themselves and they make themselves like the last priority.”* (Sarah, 34)

#### 3.3.4. Competing Priorities and Family Responsibilities

Several women identified structural and social demands, including the need to secure employment and the absence of childcare support, as significant competing priorities that constrained women’s capacity to engage with preventive SRH services.

*“You are struggling getting a job, getting money to live and then you forget about your physical health…the things that you need such as getting a job to live here. And the other thing is asylum seekers with young kids, so who is going to look after your baby if you are working. So, it’s a lot of things to be worried about because they don’t have help with child care.”* (Maya, 43)

#### 3.3.5. Self Efficacy, Motivation and Fear

A minority of women highlighted how low self-efficacy and diminished motivation, often linked to experiences of depression and anxiety, negatively influenced their willingness to engage in preventive health behaviours such as cervical screening.

*“…I have a friend who simply doesn’t care. She had 4 children, I explained to her that this is very important,…you have to do the papsmear. She said I don’t have patience for this sort of thing…because she is also suffering from depression she just says “I don’t have the patience to go through the process”* (Mariam, 43)

Fear surrounding perceived pain of the screening procedure deterred some women from seeking preventive SRH care. This, together with feelings of shame and shyness led to women avoiding health services.

*“I hate doing it, it’s painful. I don’t like anyone [indicates pelvic area]. [are you mainly scared of the pain?] yes and I’m shy. Because some women tell me it’s painful and I think two and three days there is blood. And I stopped to think for this test.”* (Iman 37)

*“…some women they feel shame, it’s not a good decision to have someone looking at you and they don’t like to do this they feel common and shame”* (Maya, 43)

Women also detailed how fear of receiving a positive diagnosis or the perceived lack of resources or support to address preventive SRH deterred some from screening or seeking health care services.

*“I think some people [women seeking asylum in her community] they are scared because if they tested, is it positive. What then? They are scared about the result. That’s why they don’t do go. Because they don’t want to risk it, if it is something that’s wrong or something that happens. What can they do about it.”* (Jen, 34)

Conversely, for some younger women, greater understanding of the benefits of prevention motivated them to seek care.

*“[Screening is for] like cancer prevention. Actually, I want to do breast screening to make sure for check. I want to know actually”.* (Akgni, 27)

### 3.4. Interpersonal Level

Theme: Fragmented interaction with primary health care

Almost all women indicated the importance of communication, trust, and cultural sensitivity in the context of accessing SRH care. Women’s relationships with their GPs and the lack of continuity of care were negatively impacted by language and sociocultural cultural barriers.

#### 3.4.1. Importance of the Relationship with Their GP and Continuity of Care

Several women identified barriers to accessing preventive SRH information. Some suggested a lack of accessible information and proactive communication about contraception from their health care provider may contribute to gaps in reproductive health care and family planning awareness.

*“For some of them [women seeking asylum in her community] maybe a lack of information because I’ve never seen that much talking about contraception in Australia. Even my GP never asked me but after I got my daughter then that doctor asked me are you planning to do any contraception like that, he asked, otherwise no one did ask from me. It’s like a very big secret, like it’s not much sharing information between too many migrant backgrounds.”* (Shiryani, 46)

Disruptions in continuity, such as a trusted doctor moving clinics, lead to a loss of trust and increased difficulty in establishing effective communication with new health care providers who may not share the same linguistic or cultural understanding.

*“…I am happy with my GP. She is good she changed she has moved to another clinic so…continuity particularly with a doctor that speaks our language, that’s important. So when they change and we get a doctor that speaks only English so it’s harder to have that relationship”.* (Jen, 34)

A minority of women reported missed opportunities to discuss preventive SRH when attending their health care provider.

*“Sometimes doctors are not talking about it. I don’t know my GP, a lady doctor but she never asked which kind of contraception…”* (Shiryani, 46)

Although some women had arrived in Australia at a time when they were eligible for HPV catch up vaccination, none reported hearing about it or receiving it. The majority of women with eligible children reported a GPs recommendation and access to trusted information enabled them to seek advice about HPV vaccination consent for their children.

*“If it’s meant to be given to them [my children] I will go to my GP who speaks Tamil and I will ask him to explain to me why it’s being given and only after I am understanding that will I give consent.”* (Vijitha, 28)

#### 3.4.2. Language, Culture and Gender Concordance

Women’s engagement with cervical screening was positively influenced by the availability of female health care providers and by concordance in language and cultural background. Women reported that cultural norms around modesty, as well as greater ease and trust in communication when speaking with female providers who shared their language and culture, were determining factors in their decision to seek care.

Availability of female providers with whom they shared language and culture was identified as an enabler of care. “Also women in my culture are shy not to go and open check her privates, this is not allowed she will not go…Well if there is a female doctor then I will go” (Maria, 36). The importance of concordance in GP-patient language and culture was also reported by several women of certain ethnic backgrounds. “Yes because all my friends are Arabic and they like Arabic doctors and women. Because the women can speak to all things, with the men doctors I think I am shy, I can’t speak [to male doctors]” (Iman, 37) and this

*“A female doctor with my language. GP female doctor and gynae doctor who is also the same doctor. [Patients initial GP] He tells me to go to the female doctor she speaks my language, screening also she helped me.”* (Maria, 36)

Theme: Spouse/family influence and support

Sociocultural cultural barriers were also reported such as women requiring their husband’s approval for decision-making, particularly when accessing contraception and cervical screening. With regard to contraception, women felt unable to fully articulate their health concerns or understand medical advice, leading to feelings of inadequacy and mistrust in health care interactions.

*“Yes, it’s kind of like taboo to talk about contraception and sex kind of thing, some of the women don’t like to talk even to their GP, they are very insecure about that…With the GP they are thinking there is not that much connection with the doctor but the nurse they [women] are a little bit closer to them.”* (Shiryani, 46)

#### 3.4.3. Partners Approval for Contraception and Cervical Screening

In some cases, women’s decisions about cervical screening and contraception were influenced by their partners’ control and approval. A few women reporting that their partners dictated the continuation of contraceptive methods, regardless of the women’s own preferences or health concerns.

*“My side what happened was that bleeding too much, it started but by one year my husband very upset bleeding going on 15 days 20 days normal so he said “OK take it down I don’t want it” [IUD]”.* (Maria, 36)

Several other women reported the negative influence of gender dynamics and cultural perceptions on the health-seeking behaviours of women, particularly in relation to cervical cancer and broader health care was highlighted by some women. The following quote illustrated this

*“Sometimes so they’re [women] not caring about their health issues, they are dependent. The husbands don’t see it as important, their wife’s health. I think maybe the GP or something, we have to educate men also as well I think. [Men] don’t know about this kind of [cervical] they don’t know fully.”* (Shiryani, 46)

#### 3.4.4. Cultural Factors and Extended Family Pressure

The influence of extended family members was identified as a significant factor affecting women’s reproductive decisions, with differing expectations and pressures from family members sometimes leading to unspoken conflicts and lack of communication between partners.

*“There is another cultural issue which is extremely important which is the interference of the extended family. [ND can you talk w a bit more about that?] The example is my own life. When I got married I didn’t want to have children right away, I wanted to have a little bit of just knowing my husband or having some fun or just not having children. My husband on the other hand wanted to have children straight away and my mother was saying don’t become pregnant soon, just take your time. His mother was saying when are you going to have the kids. I was not actually confiding to my husband what my mother was saying and he was not confiding to me what his mother was saying but it was unspoken words.”* (Sabagie, 34)

#### 3.4.5. Positive Influence of Women’s Relatives on Understanding the Need for Prevention

Enablers of care were also reported by some women and focused mainly on the positive view of understanding the need for prevention. Perception among many women that vaccination is important for personal and communal health, though they acknowledge individual hesitations or differing perspectives on its uptake.

*“That’s to protect myself and the people around me,…I want to protect my family and I get why someone didn’t do it, [vaccination] but yes I think it’s good for my health.”* (Akgni, 27)

Women’s views on seeking care were positively influenced by relatives’ and friends’ experiences. Hearing about a family member or friend’s experience of breast or cervical cancer improved women’s awareness of the benefits of screening and prompted them to seek information about or undergo cervical or breast screening.

### 3.5. Community Level

Theme: Limited community support

Cultural factors and extended family pressure, social isolation, and religious norms, operated as community level barriers to access to preventive SRH care. Women described how religious and cultural mores were maintained upon arrival in Australia, establishing rigid frameworks that limited their autonomy and decision-making.

#### 3.5.1. Social Isolation

Social isolation emerged as a significant barrier to accessing contraceptive information and services. Women noted the difficulty discussing family planning openly due to a lack of social support networks and cultural taboos surrounding the topic.

*“Well um those that want to take it [contraception] is because maybe they have come to 2 or 3 kids now and they don’t want to have anymore. And life is so hard in Australia with children, while back home where I came from I can leave my children with a friend with an Auntie without any problem but here it is not like that. So, you kind of can’t think no I cannot do this here I have to stop.”* (Afidji, 35)

Most women described social isolation and lack of community support as limiting their access to SRH information and their ability to navigate services.

*“…I came over here when I was only 19 years old…I didn’t come with my parents. If they were here I could have asked my mother… so, I didn’t know what to ask or what to go for. I wouldn’t know [why women don’t do screening] because the women from my community… that I know are much older than me, I’m much younger and I would be reluctant to ask them about whether they would like to do it [cervical screening] or why they are not doing it.”* (Vijitha, 28)

When women were asked about contraception specifically, this was a typical response.

*“Nobody talks openly about this. I don’t have much friends. We can talk because if I ask the doctor he will tell me but no talk to anyone. I don’t know anyone”* (Maria, 36)

#### 3.5.2. Religious Norms and Expectations Shared by the Religious Community

A few women openly reported how religious norms and expectations impacted their health seeking behaviour and access to SRH care. For example, in relation to contraception some women commented:

*“In my country there are some religious beliefs, it’s a Catholic country and the catholic church say you have to have the kids that God send to you so its another thing more common in the countryside.” (Maya, 43) and this “In my culture they said it is not good to use contraception, that children are a gift from God, that its not good to stop yourself from getting pregnant when you are given the chance to have children.”* (Afiji, 35)

### 3.6. Policy Level

Theme: Affordable health care

#### 3.6.1. Limited Entitlement to Medicare

Women regularly recounted examples of disrupted access to Medicare because of expiry or policy changes. Several women reported that their visa status, served as a barrier to accessing Medicare subsidised services. This was compounded by the protracted nature of their visa claims, with the majority of women facing an average wait time of eight years for resolution.

*“The [Blue] Medicare card is issued to us only for 6 months in a year. So, if we get sick family or I get sick we have to pay and see the doctors or we have to go to emergency. And going to emergency is not a nice experience either. So that’s the problem. We have Medicare only for 6 months in a year.”* (Jen, 34)

*“Well I didn’t have a Medicare for one year and I was given Medicare only 2 months ago. As I’m on a bridging visa as we are still on a bridging visa so the department of immigration is quite strict and the Medicare used to be valid for one or two years. But they are valid for only 6 months at the moment.”* (Sarah, 34) Limited access to health care due to Medicare ineligibility led to out-of-pocket costs and long waiting times for appointments. This was evidenced by women resorting to seeking hospital services instead of more suitable primary care services.

*“…after we go to the emergency or something like this, because if you want to see your GP at the ASRC you need an appointment. Some doctors don’t have any [appointments] available sometime a week, two weeks, sometimes a month, so that means we don’t have a choice we need to go to emergency…”* (Tiez, 36)

#### 3.6.2. Perceived Financial Cost

Most women were unaware that screening and vaccinations are free for eligible individuals in Australia, leading to the perception that financial barriers would prevent them from accessing these services.

*“…back home when you go for a test you have to pay out of pocket yourself, yes so its whenever you have money aside to do it then you go and do it but here its every 5 years, so people are not aware of that yes so that people who came here, if I need to go to hospital I need money out of my pocket and if I don’t have it, I can’t do it.”* (Afidji, 35)

Theme: Australia’s immigration system

#### 3.6.3. Immigration Detention

The challenges posed by Australia’s immigration system, particularly in relation to detention, were linked to women’s reproductive decisions and cease contraception. Some women attempted to conceive in an effort to avoid being sent to detention facilities.

*“I just have to tell you that for example when we were in camp there was a talk of sending us to Papua New Guinea [detention] so women were trying very hard to have children, so they would take out the Merina to have children not to be sent there [to PNG].”* (Mariam, 43)

#### 3.6.4. Visa Category and Asylum Status

Uncertainty surrounding the asylum-seeking process negatively impacted women’s contraceptive choices with concerns over visa status and the associated stress leading some to delay or cancel not only contraception use but also other necessary GP appointments. “The reason I used contraception was the visa issues. I was very uncertain about what is going to happen about the visa and also I was under a huge amount of stress” (Mariam, 43), and this “I have to cancel all of my appointments and because of my visa status because my visa is still on ongoing” (Afidji, 35).

## 4. Discussion

This study explored the experiences of women seeking asylum in accessing preventive SRH care in Melbourne, Victoria. Applying a socioecological lens, we identified barriers and enablers that intersect across individual, interpersonal, community and policy factors when accessing preventive SRH care. Seven interrelated themes were identified. These findings indicate that women seeking asylum have experienced barriers to care in part because of limited knowledge about preventive SRH care; psychosocial constraints on health agency; fragmented interaction with the primary health service; spouse/family influence and support; limited community support, affordable health care and Australia’s immigration system. Enablers of care included language, culture and gender concordance with their GP, continuity of care and supportive family members. These findings illuminate how structural and psychosocial factors impact access to preventive SRH care for women seeking asylum. These findings highlight the urgent need for context-sensitive, equity-oriented approaches improve to access to care.

### 4.1. Individual-Level Factors

At the individual level, women seeking asylum demonstrated varied levels of knowledge about preventive SRH care, with comparatively lower awareness of contraception and HPV vaccination compared with breast and cervical screening. These findings reflect broader trends observed in migrant and refugee populations, where knowledge gaps often stem from limited health literacy and lack of culturally tailored health education [37,38]. Peer influence further shaped health decisions, with misinformation about contraception, such as fears of developing cancer, infection, or weight gain, circulating within social networks and deterring uptake. Compounding these knowledge-related barriers were psychosocial constraints that significantly reduced women’s capacity to prioritise their own health. Poor mental health, shaped by trauma, displacement, and social isolation, was frequently cited as a barrier to screening participation, a finding consistent with research demonstrating the mental health effects of seeking asylum and its impact on health-seeking behaviours [39]. Competing responsibilities, including employment pressures and lack of childcare, further limited women’s ability to access care.

Feelings of fear, shame, and low self-efficacy, particularly surrounding cervical screening, and HPV vaccination, rooted in complex sociocultural factors also emerged as powerful deterrents. These findings support existing research where women are influenced by norms in their countries of origin where discussions about sexual health are often taboo and linked with moral judgment or social stigma [40,41]. From a theoretical perspective, these cultural taboos can be understood through the lens of feminist and intersectional perspectives, which highlight how patriarchal structures and intersecting cultural identities shape women’s agency and constrain conversations about sexual and reproductive health [42]. Negative attitudes toward side effects of HPV vaccination for example, may also stem from limited access to accurate, culturally appropriate information, compounded by distrust in unfamiliar health systems or past traumatic experiences with health care [43]. Shame related to HPV vaccination may be connected to perceptions that it is only relevant for sexually active women, which can conflict with community or personal beliefs about acceptable sexual behaviour [44]. These individual-level barriers illustrate how psychological stressors intersect with limited knowledge to undermine women’s autonomy and motivation to engage in preventive SRH care. Together, these findings underscore the need for comprehensive, psychologically sensitive care, and culturally responsive interventions that not only improve health literacy but also address the emotional, social, and logistical barriers that constrain asylum-seeking women’s engagement with preventive SRH care.

### 4.2. Interpersonal-Level Factors

Interpersonal relationships played a dual role in shaping women’s access to preventive SRH services. Fragmented interactions with primary health care providers emerged as a key barrier to preventive SRH care among women seeking asylum. Findings indicated that continuity of care was frequently disrupted, undermining trust and effective communication, particularly when shifting GP service provision occurred and when language and cultural concordance were lost. Similar findings have been reported in Australian studies, where disruptions in GP continuity and a lack of trust in new providers negatively impacted refugee and asylum-seeking women’s engagement with SRH care [40,45]. Additionally, women’s accounts in our study revealed limited proactive communication from providers about contraception and HPV vaccination, contributing to missed opportunities for care, a concern echoed in other research indicating that SRH is often deprioritised in general consultations with women from refugee-like backgrounds [46,47]. Conversely, strong provider–patient relationships, particularly with female GPs who shared the women’s language and cultural background, were critical enablers of SRH care. Language and gender concordance have consistently been shown to enhance comfort, trust, and disclosure during SRH consultations [48,49]. These findings underscore the importance of culturally responsive, linguistically appropriate, and gender-concordant care in fostering trust, improving communication, and enhancing uptake of preventive SRH care.

### 4.3. Community-Level Factors

At the community level, limited support, shaped by cultural norms, extended family expectations, and religious influences, emerged as significant barriers to preventive SRH care. Findings indicated the persistence of culturally sanctioned gender roles and familial hierarchies following resettlement, which often constrained women’s autonomy in reproductive decision-making. Extended family pressure, particularly from in-laws, influenced choices around childbearing and contraception, sometimes resulting in unspoken tensions between partners. These dynamics mirror findings from prior studies highlighting the role of patriarchal family structures in shaping SRH choices among refugee and migrant women [50,51]. These findings reinforce the need for culturally responsive health interventions that engage families and communities to support women’s reproductive autonomy in the resettlement context.

Social isolation further compounded the challenges associated with cultural norms and family expectations, limiting opportunities for peer discussion and access to informal knowledge networks, particularly in the absence of familiar community supports. Findings showed a reluctance to seek information or engage in discussions related to preventive SRH within religious communities, where such topics are frequently regarded as taboo [52]. This aligns with existing literature underscoring how community-level stigma and cultural silence around SRH can inhibit help-seeking behaviours [40,53]. Collectively, these findings demonstrate the need for culturally safe, community-informed strategies that foster trusted peer support and facilitate open dialogue around SRH in resettled communities of women seeking asylum.

Spouse and family dynamics also have a twofold influence on women’s engagement with preventive SRH care. On one hand, cultural expectations and gender norms, such as the need for spousal approval, acted as barriers to care, limiting women’s autonomy in decisions related to contraception and cervical screening. Findings suggested how husbands’ control over contraceptive choices and a lack of awareness or prioritisation of women’s health among male partners, contributed to delayed or forgone care. From an intersectional feminist perspective, these findings highlight how gendered power relations within families intersect with cultural and social structures to shape women’s health agency. Patriarchal norms that position men as decision-makers over women’s bodies are further compounded by intersecting factors such as migration status, socioeconomic dependence, and cultural identity, which can intensify women’s vulnerability to reproductive health inequities [54]. Within this framework, limited autonomy is not merely an individual constraint but a reflection of broader structural power imbalances that define who has authority in reproductive decision-making. Women reported negotiating health decisions within unequal power dynamics, where prioritising household stability or avoiding conflict with partners took precedence over their own health needs. Additionally, pressures from extended family members created further tensions, reinforcing traditional roles and expectations that constrained decision-making. In several cases, familial pressure to conform to cultural or religious norms around fertility and modesty discouraged open discussion of SRH needs or attendance at preventive services. A scoping review by Sawadogo (2023) identified cultural norms, including the need for spousal approval, as key barriers to SRH care among migrant, internally displaced, asylum-seeking, and refugee women [53]. The review emphasised the necessity for culturally sensitive interventions to address these challenges [53]. Conversely, family and peer networks could also serve as facilitators of care. In our study positive influence from relatives, including shared experiences of preventive care, enhanced women’s understanding of the importance of screening and vaccination. These findings align with existing literature, both in Australia and internationally, which highlights the influential role of family networks, particularly collective experiences of illness, in enhancing refugee and asylum-seeking women’s understanding and uptake of screening and vaccination [55,56,57,58]. Studies conducted in Australia also emphasise that family members can act as trusted sources of health information, reinforcing the importance of culturally tailored, family-inclusive approaches to preventive education [22]. Jointly, the contrasting experiences outlined highlight the need for family inclusive health education strategies that address both barriers to and enablers of care within familial and community contexts. For women seeking asylum, the intersection of these cultural dynamics with structural barriers outline below in policy-level factors creates additional layers of disadvantage.

### 4.4. Policy-Level Factors

At the policy level, this study highlighted experiences of structural disadvantage, including insecure visa status, ineligibility for Medicare, perceived financial cost, and limited access to appointments that collectively undermined their ability to prioritise preventive SRH care. These results are consistent with previous research demonstrating that restrictive migration policies, particularly for women on bridging visas or without permanent residency, create substantial barriers to accessing care in Australia and other high-income countries [9,59]. Moreover, the psychological impact of prolonged visa uncertainty and social isolation deprioritise preventive SRH care and increase vulnerability to poor SRH outcomes. In addition, previous studies have identified prolonged residency insecurity being linked to diminished social integration further depriving women seeking asylum of the opportunity to seek health care [60].

While this study did not primarily aim to investigate mental health outcomes, it indicated that psychological distress and mental health-related challenges in the post-migration period were significant barriers to accessing SRH care for women seeking asylum. It has been well established that the mental health of people seeking asylum is undermined by post-migration stressors, in particular prolonged immigration detention and temporary protection [61]. Half of the women in this study spent time in immigration detention, with the remainder held in community detention. Mental health is significantly worse in asylum seekers who experience prolonged detention compared with those never detained [62]. Policies that restrict access to SRH services for women seeking asylum impact health equity and increase health disparities by contributing to delayed diagnoses and unmet SRH needs [53].

In our study limited or temporary access to Medicare, often dictated by visa status and subject to sudden policy changes, resulted in delayed care, out-of-pocket costs, and an overreliance on emergency services. These findings align with prior research showing that visa-related Medicare ineligibility contributes to fragmented access to primary care among asylum seekers in Australia [25]. Compounding the limited access to Medicare were widespread misconceptions about the cost of screening and vaccination, which led avoidance of preventive SRH services women believed were unaffordable. Additionally, the uncertainty and stress associated with prolonged asylum claims, often lasting several years, were linked to missed appointments, disrupted contraceptive use, and in some cases, deliberate pregnancy as a strategy to avoid immigration detention. These reproductive decisions reflect the deep entanglement between health and migration policy, echoing findings from studies documenting how Australia’s immigration system impacts women’s autonomy and reproductive health [39]. Addressing these barriers requires policy reforms that ensure stable, long-term access to health care entitlements for asylum seekers, alongside culturally appropriate communication to clarify service eligibility and cost.

Despite recent policy improvements at both state and federal levels in Australia to enhance health care access for Medicare-ineligible populations [63,64], significant gaps remain for women seeking asylum in relation to preventive SRH care. This research reported limited access to information about cervical screening and HPV vaccination, critical components of preventive SRH care. While this study did not directly explore Medicare ineligibility, women’s experiences aligned with existing research indicating that lack of Medicare access reduces health care utilisation among asylum-seeking women in Australia [25]. These findings suggest that Medicare ineligibility impedes access to preventive SRH services, reinforcing the need for systemic policy reforms. Drawing on these insights, we outline multi-level recommendations to improve access to preventive SRH care for women seeking asylum (Table 3).

The study’s’ strength is that it provides valuable insights into the lived experiences of women seeking asylum, a group often excluded from health research and policy discourse. By centering women’s voices and using a socioecological framework, the study highlights both structural constraints and opportunities for agency within the health system. The study was strengthened by recruitment of women seeking asylum from a range of support agencies, together representing eight countries of origin. For women with limited English language skills interviews were conducted with a NAATI accredited interpreter allowing them to fully express their views.

However, the findings should be interpreted in light of several limitations. The sample, while diverse in region of origin, was limited to women engaged with a community-based service in Melbourne and may not reflect the experiences of those more socially isolated or in other settings. Although multiple recruitment strategies were employed, including outreach through services, professional networks, and word of mouth, these approaches may not have effectively reached women who were truly ‘hard to reach,’ such as those not engaged with services or social networks. Most women learned about the study through a service provider or community leader, indicating some level of connection with local organisations. Broader and more sustained community outreach efforts may have facilitated participation from women beyond those already linked to health or community services. Despite the use of interpreters, language and cultural barriers may have constrained the depth of data collection in some cases. Conducting research with participants who have limited English proficiency and diverse cultural backgrounds presents methodological challenges, particularly in ensuring that the meanings of participants’ responses are accurately captured [65]. This process carries an inherent risk of misinterpretation or loss of contextual nuance during translation and interpretation. To minimise this risk, any ambiguity or uncertainty that arose during the interviews was immediately clarified with the interpreter, and post-interview debriefing sessions were conducted to discuss and resolve potential misunderstandings or culturally specific expressions. The use of a convenience sample may limit the representativeness and contextual diversity of participants, thereby reducing the transferability of findings to other settings or populations. This limitation was mitigated through the inclusion of women from diverse of countries of origin, across a wide age range all with markedly different immigration experiences and consider the diversity of women is a strength of this study.

While women appeared comfortable and open when discussing sensitive SRH topics, it is important to acknowledge that those who chose to participate may have been more willing to engage in such discussions. This self-selection introduces a potential risk of response bias, as the experiences of women who were less forthcoming or hesitant to discuss SRH may not be fully captured. Nevertheless, the candidness observed during interviews suggests that response bias due to the sensitivity of the topics was unlikely to have significantly influenced the findings.

Information on visa type was not specifically collected from women; therefore, distinctions between different visa categories could not be systematically examined. However, during the interviews and subsequent thematic analysis, it became apparent that visa status itself functioned as a barrier to care. Participants described how the uncertainty and restrictions associated with asylum-seeking visas influenced their access to, and engagement with, SRH services. Future research might explore the effectiveness of culturally tailored, family-inclusive, and policy-sensitive interventions to improve preventive SRH care access among women seeking asylum, particularly across different visa categories.

Future studies might examine the extent to which specific sociodemographic and experiential factors, such as duration of immigration detention, length of residence in Australia, and country of origin, shape access to SRH services among women seeking asylum. Research might explore how periods of detention constrain opportunities to engage with preventive SRH care, or exacerbate psychosocial stressors that influence health-care-seeking behaviours. Similarly, shorter durations of residence in Australia may be associated with limited familiarity with Australia’s health care system, restricted knowledge of available services, and reduced social support networks, thereby creating additional barriers to SRH access. Further research is needed to explore cultural norms and practices pertaining specifically to countries of origin for women seeking asylum to help understand barriers to and enablers of SRH access. Investigating the interplay of these factors could inform the development of targeted interventions and policies aimed at enhancing SRH equity for women seeking asylum.

**Table 3:** Potential strategies for strengthening access to preventive sexual and reproductive health care for women seeking asylum.

Approaches to Enhancing Access to Preventive Sexual and Reproductive Care for Women Seeking Asylum are Presented for Each Level of the Socioecological Model. It Is Important that Strategies to Enhance Access Occur Across the Range of Levels, with Attention to Maximising Benefits Between the Different Layers of Disadvantage.
*Individual level*
provide comprehensive education on preventive SRH care and services in the Australian context tailored to the specific cultural and linguistic needs of women seeking asylum to improve engagement with services
*Interpersonal level*
encourage GPs to consider screening for psychosocial needs and referral to asylum specific mental health support for women
encourage GPs and nurses to opportunistically inform women that cervical screening and HPV vaccination is fee free for eligible women GPs and nurses might incorporate routine SRH discussions during appointments, provide written or translated resources and use trained female interpreters when needed
consider educating health and support staff on the policy directives [58] regarding services available for Medicare ineligible asylum seekers
encourage GPs and nurses to implement follow-up care and call reminders ensuring women receive continuity of care and reduce fragmentation provide specific training for GPs and nurses in trauma-informed care
*Community level*
ensure cultural safe and competent care training reaches providers caring for women seeking asylum, including understanding women’s religious beliefs, spousal influence and encourage peer support
strengthen linkage between primary health care services and local asylum seeker support and resettlement agencies to identify hard to reach women requiring cervical screening tests, breast screening and HPV vaccination and information.
consider the establishment of ongoing health care provider led community-based health education sessions at schools, public libraries and community health services expand and enhance state-funded community health programs specifically for asylum seekers such as the Asylum Seeker Resource Centre and Victoria’s Refugee Health Program.
*Policy level*
expand resettlement support services including case manager support and provision of comprehensive health assessments for women seeking asylum [1]
ensure care is offered without financial barriers to asylum seekers by considering providing women seeking asylum with full Medicare eligibility equal to that received by refugees
consider extending the Adult Migrant English Language Program (AMEP) [66] to allow women access to 510 h of English language classes to promote independence and reduce social isolation

## 5. Conclusions

New insights are offered into the barriers to and enablers of care for women seeking asylum in terms of accessing preventive SRH care and services following arrival in Australia. Access to care in Victoria remains limited for some women seeking asylum, likely due to the influence of multiple, intersecting social, community, and policy-related factors. Using a socioecological framework and a qualitative approach, this study provides rich and insightful findings that deepen understanding of the complex interplay of individual, interpersonal, community, and policy factors affecting health care access. The study reveals opportunities to enhance women’s access to preventive SRH care and engagement with primary health care services. Conducting research on the unique needs of women seeking asylum is essential for ensuring policies and services effectively address their health needs and promote health, safety, and well-being.

## Figures and Tables

**Table 1:** Participants’ sociodemographic characteristics.

	Women (*n* = 12)
***Country of birth***	
Iran	3
Sri Lanka	3
Colombia, Indonesia, Lebanon, Malaysia, Togo and Pakistan	6
***Marital status***	
Married	11
Widowed	1
***Maternal status***	
Have children	11
Have no children	1
***Level of completed education***	
Secondary education (Year 9 or above)	7
Tertiary/university	5
***Employment***	
Home duties	10
Employed	2
***Years in Australia***	
1–5	3
6–10	8
11–15	1
***Time in immigration detention***	
Yes (2 to 6 months)	5
No	7

**Table 2:** Individual participant characteristics (*n* = 12).

Participants (Pseudonym)	Age (Years)	Years in Australia	Region of Origin	Occupation	Level of Education Completed	Immigration Detention
Afidj	35	6	West Africa	Home duties	Year 12/University	No immigration detention, arrived on a tourist visa
Maria	36	3	South Asia	Home duties	Year 12	No immigration detention, arrived on a tourist visa
Maya	43	4	South America	Medical doctor	University level	No immigration detention, arrived on a tourist visa
Sarah	34	9	Middle East	Home duties	Year 10	3 months—detention centre
Shiryani	46	14	South Asia	Home duties	University level	No detention following Ministerial Intervention
Mariam	43	10	Middle East	Home duties	Year 9	2 months—Christmas Island detention 2 months—Perth detention centre
Sabagie	34	9	Middle East	Home duties	University level	3 months—Darwin detention centre
Tiez	36	8	Southeast Asia	Home duties	Year 12	No immigration detention, arrived on a tourist visa
Akgni	27	2	Southeast Asia	Home duties	Year 10	No immigration detention, arrived on a tourist visa
Iman	37	10	Middle East	Home duties	University level	No immigration detention, arrived on a tourist visa
Vijitha	28	9	South Asia	Home duties	Year 11	3 months—Christmas Island detention, 6 weeks—Darwin detention centre
Jen	34	9	South Asia	Elderly care/personal carer	Year 12	3 months—Christmas Island detention, 6 weeks—Darwin detention centre

## Data Availability

The data are available upon reasonable request from the corresponding author.
